# Experiences of living with idiopathic pulmonary fibrosis in relation to physical activity - “How the hills became steeper and steeper”: a qualitative interview study

**DOI:** 10.1186/s12890-024-03064-z

**Published:** 2024-05-23

**Authors:** Anna Jernås, Monika Fagevik Olsén, Emma Holmqvist, Jenny Danielsbacka

**Affiliations:** 1 Department of Physical Therapy, Sahlgrenska University Hospital, Vita stråket 13, Gothenburg, 413 45 Sweden; 2 Department of Health and Rehabilitation/Physiotherapy, Institute of Neuroscience and Physiology, Sahlgrenska Academy, University of Gothenburg, Box 430, Gothenburg, 405 30 Sweden

**Keywords:** Dyspnoea, Fatigue, Function, Fibrosis, Pulmonary, Physical activity

## Abstract

**Introduction:**

Idiopathic pulmonary fibrosis (IPF) is a progressive disease presenting with symptoms like dyspnoea, dry cough, and fatigue, which affect physical function and quality of life. No earlier qualitative studies have investigated physical activity in IPF. This study aims to explore experiences of living with IPF in relation to physical activity.

**Materials and methods:**

Qualitative interviews were conducted with 14 participants living with IPF. The participants were 77 years old (range: 56–86) and diagnosed with IPF between 2 and 9 years ago. The analysis was performed by qualitative content analysis according to Graneheim and Lundman.

**Results:**

The results indicated that life and one’s ability to be physically active is affected by IPF. Despite this, it seems possible to navigate past obstacles, which was illustrated by an overall theme: “My life is constrained, but I am hanging on”. Two major categories cover topics of IPF being a life changing diagnosis with changes in self-image and changed future plans regarding physical activity, as well as life. Physical activity was perceived to be challenging, yet in many ways used as a strategy, developed to manage life.

**Conclusions:**

IPF affects physical activity as well as life, from onset onwards. By developing strategies for facilitating physical activity as well as identifying barriers, it seems possible to maintain an active life despite the disease. The healthcare system needs to create support systems that meet different needs during different phases of the disease.

**Trial registration:**

“FoU in Sweden” Research and Development in Sweden (id: 227081).

**Supplementary Information:**

The online version contains supplementary material available at 10.1186/s12890-024-03064-z.

## Introduction

Idiopathic pulmonary fibrosis (IPF) is an interstitial lung disease with unknown aetiology [1]. It is a rare disease, with an increasing global incidence [1]. The incidence among women is 13.2/100 000 and 20.2/100 000 in men worldwide, with an average age of onset/ diagnosis of 66 years for both sexes [2]. Life expectancy is median 3–5 years after diagnosis [3].

Pulmonary fibrosis causes general lung inflammation and subsequently lung parenchyma fibrosis [4]. The lungs reduced compliance due to fibrosis, in combination with the lung gaining elastic resilience, is the underlying cause of patients’ experiences of dyspnoea [5]. IPF is a chronic, progressive disease, and the fibrosis is irreversible [6]. Currently there is no cure, however, treatment with corticosteroids and immunosuppressive medication can slow down the process [6]. Also, anti-fibrotic medication can slow down decline in lung function and reduce risk of acute respiratory deterioration, which are associated with very high morbidity and mortality [7]. Lung transplant is the only treatment option that prolongs life expectancy, and it is an option for a minority of patients due to several contraindications connected to eligibility for the procedure [6, 8].

Living with IPF means living with limitations and symptoms such as dyspnoea, fatigue, impaired physical capacity, and reduced quality of life as a result of reduced lung volume, reduced oxygen uptake and dry cough [9]. Experiencing dyspnoea and shortness of breath due to IPF has been shown to affect life considerably creating feelings of losing one’s former life and independence [10]. At present, smoking cessation, oxygen treatment and, depending on which hospital the patient is connected to, general physical activity under the guidance of physiotherapists, is offered to these patients [11]. Physical activity is defined as “*any bodily movement produced by skeletal muscles that results in energy expenditure*” [12].

In a study by Badanes-Bonet et al. [13] muscle strength and psychological factors were identified as predictors of physical activity in IPF. The study concluded that there is a need for early inclusion in rehabilitation programmes [13]. It is important to capture the experiences of patients living with IPF concerning physical activity to be able to develop the optimal management of physical activity in patients with IPF. There are, to our best knowledge, no studies focusing on the impact of IPF regarding physical activity.

The aim of this qualitative interview study was to explore the experiences of living with idiopathic pulmonary fibrosis and its effect on physical activity in everyday life.

## Materials and methods

In this qualitative interview study, content analysis according to Graneheim and Lundman were used for analysis [14]. The participants were recruited from the outpatient lung clinic at Sahlgrenska University Hospital, Gothenburg, Sweden. The outpatient lung clinic have a fibrosis team with specialised respiratory physicians, a specialised nurse, physiotherapist, occupational therapist, dietician, and counselor. Patients with IPF have yearly appointments with the team. All patients are referred for contact with a physiotherapist to discuss physical activity and respiratory symptoms due to IPF. The participants were included in the study by strategic sampling among patients from the clinic by a research nurse, since qualitative research searches for as broad experiences as possible [14]. We aimed to include both male and female patients, patients with shorter as well as longer duration of symptoms, in different stages of the disease and receiving antifibrotic medication or not. Recruitment commenced in August 2017 and continued until the first 10 participants were included. All patients asked to participate accepted inclusion in the study.

All participants received oral and written information and gave their written consent before inclusion in the study. In a situation where information was considered too sensitive or private after the completion of the interview, the participant had the right to refuse publication of these segments in the study. The Ethics Review Board of Gothenburg approved the study (Ref number: 001–15). The study is registered in the database FOU i Sverige (R&D in Sweden) with the registration number: 227.081.

The initial data collection was made through individual semi-structured interviews between September – December 2017. The interviewers (AJ + EH) presented themselves as physiotherapy students at the time for inclusion. The interviewers had no previous relationship with the participants. One interview per participant was performed. An interview guide with four areas of focus was used during the interviews: Physical activity, activity/participation, quality of life and environmental factors. Open follow-up questions were formulated to cover the topics and at the end of the interviews, control questions were asked in case something needed to be added or clarified. The interview guide was tested in two pilot interviews before the study started. The participants could choose whether the interview would be conducted at their home or at the hospital’s physiotherapy department. In total, nine of the interviews were performed at the physiotherapy department and 5 in the participants´ home. The interviews lasted between 17 and 55 min. They were recorded by a recording application on a mobile phone and a tablet. Recorded data was transcribed verbatim by the interviewer in direct connection with the interview.

The material was analysed by an inductive, descriptive approach through qualitative content analysis according to Graneheim and Lundman [14], see Table 1. For this method of qualitative analysis, two theoretical assumptions are stated: reality can be interpreted in various ways and understanding depends on subjective interpretation by the researchers, and also that interviews are shaped through interaction between the participant and the researcher since ‘one cannot not communicate’ [14]. The interpretation made during the analysis is influenced by the researchers pre-understanding, meaning that the experiences and qualifications the researcher has is important [14].The inductive approach implies that the analysis is data-driven and characterised by searching for patterns within the text [14].

**Table 1:** The analysis process of qualitative content analysis according to Graneheim and Lundman

1. The text was read through several times to create a sense of wholeness.
2. Topics congruent to the aim were marked in the interviews, i.e. the unit of analysis.
3. The marked text was then divided into meaning units.
4. The meaning units were condensed, i.e., the sentences were shortened without changing the meaning.
5. The condensed text was abstracted which means that the content and interpretation of the condensed text was described at a higher level of abstraction in a code.
6. These codes were then sorted based on similarities and differences. The codes were divided into categories and sub-categories.

When the initial ten interviews were completed, analysis commenced. The analysis and coding were discussed among the four authors until consensus was reached. Based on the analysis of the first ten interviews, saturation of data was not reached, and additional participants needed to be included. A new recruitment period started in October 2018 to complete the study group and were performed by the same research nurse. All four patients asked to participate accepted. The additional four interviews were performed by AJ, then registered physiotherapist, during November 2018 - February 2019, and new topics were found in the interviews, i.e. the unit of analysis. After completing the analysis of the four supplementary interviews, saturation was fulfilled. All four authors are female physiotherapists with currently 5 to 37 years working experience and two (JD and MFO) are clinical respiratory specialists. Consolidated criteria for qualitative research (COREQ) were used to enhance the trustworthiness of the whole research process, including analysis and presentation of the results.

## Results

Fourteen patients diagnosed with idiopathic pulmonary fibrosis (IPF) were included in the study, see Table 2. The participants physical activity levels varied between being able to walk 50 m with a walker or being able to go for a shorter walk, to training at a gym several times a week or swimming. None of the participants were part of a pulmonary rehabilitation set up, however, all participants had contact with the physiotherapist at the outpatient lung clinic.

**Table 2:** Demographics of included participants, *N* = 14

Female (*n*)	< 5
Male (n)	11
Age, median (range)	73.5 (56–86)
Years since diagnosed with IPF, median (range)	4.5 (2–9)
Antifibrotic medication, (n)	8
Oxygen treatment, (n)	< 5
FVC %, median (range)	93.5 (74–122)
Diffusion capacity %, median (range)	43 (25–60)
Smoking, (n)	< 5
Living with another adult (n)	11
Working (n)	< 5

*IPF* idiopathic pulmonary fibrosis

**Fig. 1:**
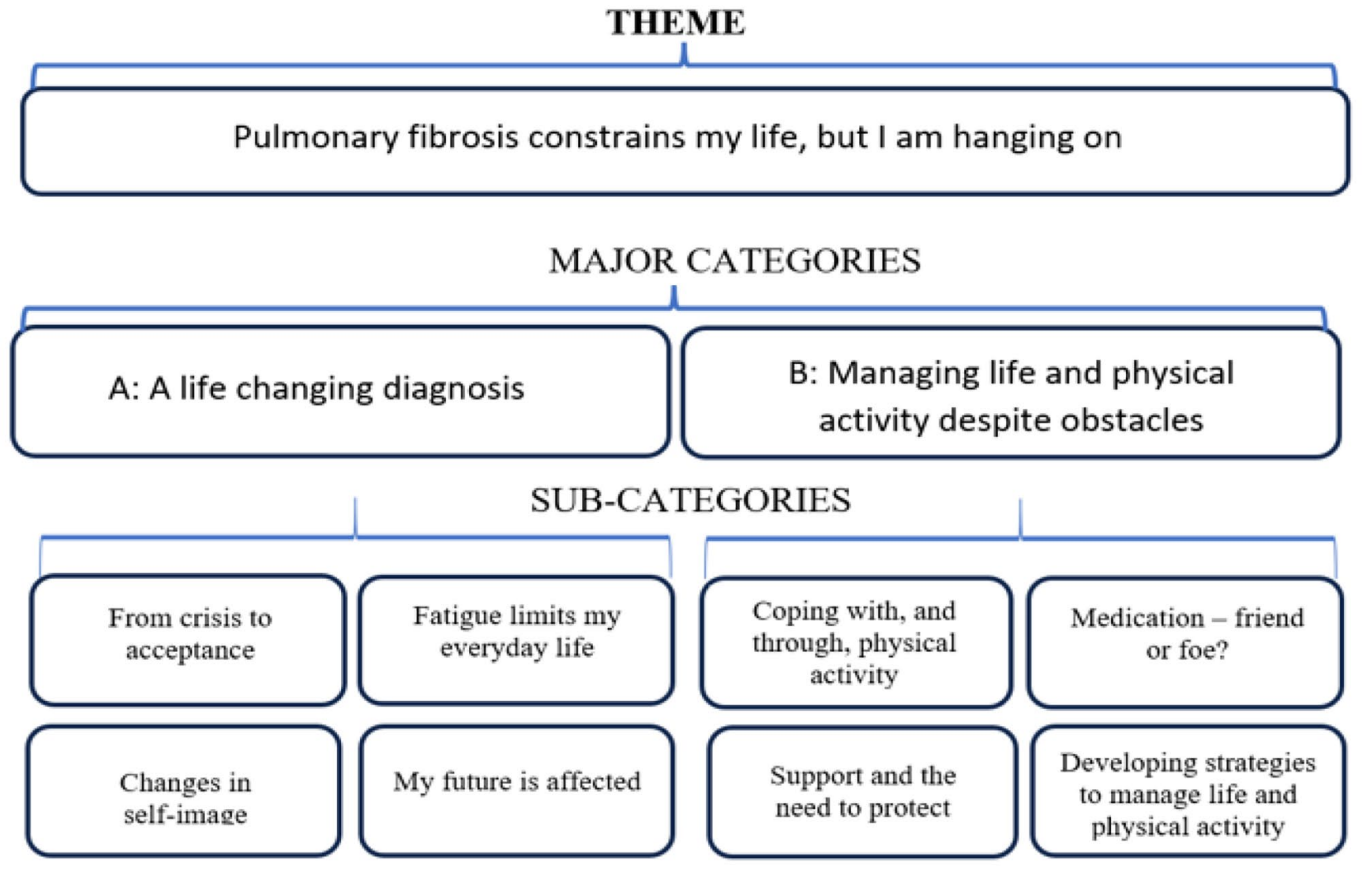
Overview of theme, major categories, and sub-categories

An overall theme was identified in the unit of analysis: “Pulmonary fibrosis constrains my life, but I am hanging on”. The theme was apparent in all four foci of the interview. Connected to the theme, two main categories were found. Major category A; “A life changing diagnosis” and major category B: “Managing life and physical activity despite obstacles”. Each category had sub-categories related to them as shown in Fig. 1. The theme, the major categories and sub-categories are presented below and illustrated with quotes from the participants. To be transparent regarding the quotes, the number of the participant is listed at the end of each quote.

### The theme – “Pulmonary fibrosis constrains my life, but I am hanging on”

Despite obstacles described connected to the diagnosis that affected and constrained their lives and their ability to be physically active; the participants expressed small but clearly presented glimpses of hope in between the struggles of everyday life. Throughout the difficult experiences associated with trying to live a physically active life, there were also experiences that stood in opposition to them. These experiences stood for resistance against their minds and bodies being taken over by the diagnosis and the hardships that come with it. Being diagnosed with a life-threatening disease evoked many feelings, both burdensome but also accepting and a willingness to find strategies to cope with physical activity in this new life.

### Major category A: a life changing diagnosis

This major category contains experiences of being diagnosed with IPF which meant that life changed from a usual everyday life to a new life where matters such as crisis management, changes in self-image regarding physical activity, and symptoms affecting many aspects of life were needed to be dealt with. The new diagnosis brought thoughts of what was to come in the future, about the progression of the disease and how that will affect their lives and their ability to be active. This category comprises four sub-categories: *“From crisis to acceptance”, “Fatigue limits my everyday life”, “Changes in self-image” and “My future is affected”.*

#### From crisis to acceptance

When being diagnosed with pulmonary fibrosis the information about life expectancy, respiratory symptoms with following physical limitations, treatments, and interventions were perceived as scary, and shocked the participants. Descriptions of distress related to existential thoughts about the progression of the disease and life expectancy were expressed. Following the diagnosis, a crisis response with feelings of considerable fear were experienced, which took a lot of energy and had a negative impact on life. The participants described that they were psychologically affected by the irreversibility of IPF and its effect on respiratory function, affecting many parts of their lives. This psychological effect caused them to feel more depressed in periods. Different aspects of life, such as the forthcoming limitation of their ability to be physically active, were described as facts they had to accept. On the other hand they were having difficulties actually accepting their new life situation. Trying to come to a place of acceptance regarding their diagnosis of IPF was very commonly overwhelming. Acceptance of having a disease without understanding the underlying cause/s was described as difficult. The participants described needing to find acceptance, not only for their current physical function, but for their life going forward and their disease.

*“When I got home after receiving this diagnosis, I could not even spell ´pulmonary fibrosis´. I started to google, and when I did, I just pulled the power cable out of my laptop straight away, because there was nothing fun in what I was reading”*.

Participant 4

*“After finding out all that I thought “well, something is gonna kill you”. So, you don’t sit and be bitter about it…there are worse fates, that’s how I think about it.”*

Participant 2

#### Fatigue limits my everyday life

Fatigue and an increased need for rest during their daily life and during physical activity was described by the participants. This new need for rest led to a constant experience of limitation and stress towards general activities, participation, and physical activity in their everyday lives. The participants described that fatigue also affected their lives, making them lose their spark/interest in activities and hobbies that before the onset of IPF were meaningful and gave energy, such as fishing, dancing, and socialising with friends. One description of fatigue was the feeling of having a wet blanket covering them during activity, creating feelings of limitation towards activity. This limitation meant that every activity required more energy than previously and that also affected them mentally. The extra energy expended reduced their willingness or interest in performing activities they were used to doing, as well as stopping them from pursuing new activities. The need for rest was expressed as constant, even during days with few activities at a low pace. Symptoms such as fatigue and drowsiness negatively affected participants’ reported quality of life.

*“On the other hand, fatigue, I feel like I’m so much more affected by it and I get tired and need to rest more.”*

Participant 8

*“I just have to accept that I, when I’m in the garden and working, digging and cleaning and taking care of the fishpond, I can work for two hours, then I get to rest. Half an hour at least. And, if I do that, it works. Then I can start again for a while”*.

Participant 6

#### Changes in self-image

The participants described a changed view of themselves where they have revised the self-image from healthy and physically strong with energy, to someone weak with a chronic progressive disease. Despite this, the participants did not feel that relatives and other people around them thought differently about them after the onset of illness. Feelings of finding it hard if other people in their environment perceived them as impaired or altered in capacity, led them to avoid social interactions in order to not be ashamed of their impaired function. Experiences of a reduced desire and willingness to do things that they previously did without feeling resistance were acknowledged. This reduced desire was described both in relation to a reduced physical ability, such as sexual function, but also to a mental influence leading to an increased reluctance in general.

*“And I think it’s embarrassing. Mmmm. Really embarrassing. / … / It has gotten so much worse that I don’t want to go out walking with friends. My children and grandchildren are well aware of my situation, so I don’t think that that is hard. And my close friends, you know, they have to stop walking when I do. But, my friends who don’t know, then I avoid walking with them, I’ll find something else to do, or go on like that. If we were going to be going for a walk together.”*

Participant 1

*“Yes, so you can talk about private stuff too, so it affects living together as a couple too.”*

Participant 8

#### My future is affected

The participants described an uncertainty about their future or how long their current physical function will be preserved, and how quickly the physical deterioration will occur. It was formulated that they did not plan as far ahead as before, depending on how life will turn out. Thoughts about ageing and the course of nature and how life could also be limited by other potential illnesses or injuries were acknowledged. A willingness to continue living a physically active life were expressed and the participants described having more things they wanted to do in their lives before they were further impaired in their physical function. The participants related their planning to the course of the disease and described the planning as a reluctance of giving in to the disease. At the same time, they expressed an awareness since their disease will not improve significantly over time. Wanting to avoid the need to constantly live with oxygen therapy during physical activity was expressed, as well as thoughts of an impending lung transplant and its impact on the future were acknowledged.

*“So… last year, I bought, or I booked the trip, // …because we go up and fish, for the summer.// And I thought when I booked it, that I’ll never get there. But. I booked it anyway. I didn’t want to disappoint my children. To disappoint my son. /…./ And now I booked it again this year. But there were worries last year. It was hard, actually. But it worked out okay in the end.*

Participant 12

*“But overall, I can’t complain about my life, even if I find it hard to keep up with as much as I want to. That is so hard to find time for I’m afraid. / … / So, that’s kind of a bit of a struggle between having the energy for, or time for it actually”*.

Participant 5

### Major category B: managing life and physical activity despite obstacles

In this major category different sides of life and management of physical activity, regarding coping with being diagnosed with idiopathic pulmonary fibrosis were explored. Experiences of physical and mental struggle connected to relationships with others and how to manage physical activity were acknowledged. Finding ways to get through life with all its activities, even if IPF affected so many aspects of everyday life, by finding sources of strength in the midst of one’s daily battles. The category contains four sub-categories: “*Coping with, and through, physical activity”, “Medication – friend or foe?”, “Support and the need to protect others”* and *”Developing strategies to manage life and physical activity”.*

#### Coping with, and through, physical activity

The first signs of limitation of physical function, were experiences of poorer physical fitness or an increase in respiratory infections. Coughing and dyspnoea were the symptoms described as affecting physical activity most. A common physical limitation mentioned was that the ability to walk uphill was suddenly more demanding. Another form of physical activity described as affected, was the participants’ ability to perform sexual activities due to respiratory symptom limitations. The participants described that they had to be mentally strong, to force themselves to be motivated, when physical activities were demanding. By setting up their own different goals, eventually motivated by contact with the physiotherapist, with their exercise and practicing different types of setups, amounts of activity, and intensity, as well as trying to adapt their physical activity along the course of the disease was used to increase motivation. A positive part of healthcare treatment experienced by the participants was contact with a physiotherapist. The physiotherapist focused on their resources with the goal to increase physical capacity. The contact with physiotherapists gave the participants motivation to maintain physical activity and also strengthened their physical capacity, which was perceived to slow down the disease progression and relieve symptoms. The experience of physical activity, whether it involved physical exercise or everyday activities, was mixed. However, the participants found an increased well-being from physical activity, despite reduced functional capacity and endurance. The participants acknowledged that they often had feelings of reward when doing well.

*“Er, and then I started coughing more and more and my training got, after the training sessions, I had huge coughing fits and the following day in the morning and during the day I had coughing fits. It was just a dry cough, a dry cough.”*

Participant 12

*“The hills where I live, it’s very hilly, they became steeper and steeper.”*

Participants 4

*“I usually say that at that point, I’m the fastest statue in the world. I hardly move. Because it takes such a long time.”*

Participant 6

#### Medication – friend or foe?

The participants had different experiences about the medication that aims to slow down the progression of the disease and the destruction of pulmonary tissue. Different side effects, such as intense gastrointestinal symptoms, for example extensive diarrhoea, restricted their participation in physical activity. The gastrointestinal symptoms could be experienced as so troublesome by some participants, that it made them to no longer want to take the medication. On the other hand, other participants had experiences with the medication resulting in good effects on the disease, thereby improving their quality of life, and allowing them to participate in different types of activities that were earlier restricted.

*“So when I went back… I told the doctor there that I don’t want this medicine anymore. That I have decided that I would rather die six months earlier. And feel like I have a reasonable quality of life.“*

Participant 6

*“And it has a very good effect on this. It suppresses it. Yes, I want to say that it increases my quality of life, without it I had, it was much, much worse, (without it) then I would have had a lot worse time in my everyday life.”*

Participant 8

#### Support and the need to protect others

The social support the participants felt from relatives since they fell ill, in the form of commitment and willingness to help when their physical function declined, was something that they experienced as a strength. The experience was that close relatives showed understanding of the disease and its consequences, such as reduced physical function and variation in mental health. Understanding work colleagues made it easier to keep attached to one’s workplace even though work itself was demanding both mentally and physically. The participants described their involvement with family and friends, where experiences of being able to participate in significant and close relationships as satisfying. However, the participants also described problems concerning communicating and interacting with close relatives, since they had feelings of trying to protect their relative from how affected by the disease they actually were.

*“It’s her, my wife, who keeps me alive. Otherwise, I don’t think I’d be here. I… clocked out voluntarily”*.

Participant 12

"*There’s been a lot of trouble with my wife and me. I haven’t talked about my stuff so much with her, I don’t want to share my, my misery with her then, but maybe you should do that more*."

Participant 13

#### Developing strategies to manage life and physical activity

The participants described how daily physical activities had to be adapted to being performed without too much limitation or being too costly related to physical and mental stress. Descriptions of the need to map out and plan physical activity, for example by choosing roads while walking, avoiding hilly terrain. Setting goals to avoid hills and walks that were too long, as well as stressful social situations was described. Regarding their perceived physical and mental function, the participants indicated that they tried to focus on what they could do and not on their limitations. The participants used their car to a greater extent or went by public transport to avoid excessive walking. Another strategy was to keep psychologically and physically busy by taking care of the house, so as not to have time to think too much about how emotionally heavy it could feel at times, as well as keeping physically active.

*“So I buy food online, I don’t have to carry it at all.”*

Participant 1

*“Previously, I might have gone shopping and walked home and stuff, but now I take the car.”*

Participant 2

## Discussion

In this qualitative interview study, with the aim of exploring living with idiopathic pulmonary fibrosis (IPF) in relation to physical activity, an overall theme was identified. The theme: “Pulmonary fibrosis constrains my life, but I am hanging on”, has two major categories with related sub-categories attached. The theme covers experiences of IPF being a life changing diagnosis leading to a life suddenly full of obstacles in need of being conquered. However, ever present in the results was the desire to somehow overcome these obstacles. The results indicate that IPF, from time of diagnosis, confers both a mental and physical effect. This was perceived to inevitably affect the possibility to be as physically active as before, as well as changing the course of the future.

The participants described that their physical function had already deteriorated early in the onset of their IPF. Physical activity evoked feelings of being hard to perform and endure because of symptoms such as fatigue, dyspnoea and cough that were connected to it. But physical activity also gave feelings of reward, as it was perceived by participants to slow down the course of the disease and relieve symptoms. These dualistic feelings shone through both the participants’ description of physical activity, as well as their description of life in general after diagnosis. The same form of duality is described by Brown [15] in a study on patients with oncological diagnoses and the impact of hope in chronic disease. Hope is defined by Brown as a necessary and essential response to hardship to overcome adversities common at times of crisis [15]. In Brown’s [15] study it became evident that the patients experienced a paradox between keeping their hopes high enough to embrace the potential effects of a new treatment, versus avoiding hoping for too much and ending up in despair. Patients with IPF need to be given the opportunity to keep up hope of a life where energy and the identity of being an active person is resumed through performance of physical activity. The healthcare system needs to facilitate opportunities for patients with IPF to be able to perform physical activity, thus giving the patients a way to maintain hope of an active life despite their diagnosis.

Many obstacles were described by the participants regarding their possibility or willingness to be physically active. Fatigue was one symptom described as debilitating, both with regards to physical activity as well as activities in need of mental effort. Changes in self-image, that transforms oneself from a physically strong and capable person to someone with a chronic disease was also described as a reason to avoid activities with other people. Despite this, finding ways to facilitate physical activity and everyday life activity in general was acknowledged as a positive thing by the participants. By saving energy by doing activities with less energy expenditure, allowed the participants to keep control of their physical activity. So even if physical activity was perceived as being forever changed, it could still be managed through different coping strategies. Kristofferzon et al. [16] found that coping strategies were important factors to establish a relationship between quality of life and coherence in patients with chronic illness such as heart failure, end-stage renal disease, multiple sclerosis, stroke, and Parkinson. Kristofferzon et al. [16] writes that those who are able to identify and use their own general resistant resources have a stronger sense of coherence and perceive better quality of life. In a scoping review by Lee et al. [17] self-management is highlighted as different factors that can enhance the patients´ self-efficacy towards being able to manage their IPF. The need for support through, for example: learning about coping strategies, patient education, psychosocial support, and home exercise were pointed out as important parts of self-management [17]. The ability to be able to create coping strategies and management of disease can be difficult alone, and this further enhances that patients with IPF need a support system that meets their different needs during different phases of the disease.

The participants in the present study described that they were psychologically affected by the irreversibility of their disease and that the forthcoming limitation of their physical abilities were acknowledged as a concern. However, the participants expressed that one part of the healthcare treatment they experienced as positive was the contact with a physiotherapist. Through the physiotherapist focussing on the participants resources, they received motivation to maintain physical activity despite the obstacles connected to it. Somogyi et al. [6] states that during the development of treatment options, it is important to improve therapies that are non-pharmacological such as rehabilitation and management of symptoms. Duck et al. [10] performed a qualitative interview study with the aim of exploring experiences and needs of patients with IPF. The participants expressed that the disease, through its rapid deterioration, gave them too little time to adjust and cope with their new life [10]. They experienced having IPF as having an “unseen” disease and sought more support from the healthcare system and perceived the support structures around IPF as lacking in contrast to, for example, cancer care [10]. Mendes et al. [18] reviewed exercise-based pulmonary rehabilitation for patients with IPF and found that developing rehabilitation programmes suitable for these patients is a complex task. It is known that exercise-based rehabilitation in interstitial lung disease is beneficial for improvements in six-minute walking distance and by lower subjective assessments with respiratory burden questionnaires [19]. However, Mendes et al. [18] identified barriers and facilitators important to be aware of, such as the patients not knowing the benefits of the rehabilitation programme, or the patients’ family being fearful concerning their relatives physical and emotional limitations which could colour the patients’ experience of the programme negatively. Facilitators include strategies to improve information given to the patients to assure they understand the benefits of the rehabilitation programme, as well as educating healthcare professionals of the importance of attendance to the programme, which could make them more persuasive in their attempts to recruit patients to the rehabilitation programmes [18]. Mendes et al. [18] stated that since barriers and facilitators of rehabilitation include physical, social, psychological, and motivational subjects it means that pulmonary rehabilitation for patients with IPF should be integrated with other care. In relation to the results of the present study it is clear that IPF affects many parts of patients’ lives, meaning that they need team-based care where they can receive support and learn about self-care management to be able to cope with different aspects of life and physical activity.

### Strengths and limitations

In qualitative research it is important to be transparent regarding the analysis process to be able to reach trustworthiness of the results. There are several ways to increase trustworthiness, for example by looking at the credibility, dependability, and transferability of the research process as well as the results [14]. In this study credibility is enhanced by the variability of the participants age, gender, and time since diagnosed with IPF. This provides conditions to reach a broad perspective and richness of experiences and perceptions regarding the research questions. The analysis was performed by all authors separately initially, and furthermore in collaboration to reach consensus regarding the results. The results with the major categories and connected sub-categories covers the whole material well leading to the formation of an overall theme. Two of the authors (EH and AJ) performed the interviews and by the use of a semi-structured interview guide that assured that all participants got the same questions, enhances the dependability of the results. Whether the results are transferable to other populations with IPF, or other interstitial lung fibrosis is not for the authors to decide. However, by having a transparent description of the execution of the study regarding settings and participants, as well as for the analysis process, and by the use of quotes directly transferred from the participants, transferability is enhanced, yet it is for the reader to decide. All interviews were performed before the COVID-19 pandemic. The impact of the pandemic is therefore not known. However, in a qualitative study where approaches during the pandemic was investigated in Australian patients with ILD it was found that the patients had different self-management and monitoring strategies that were key to managing the impact of the pandemic, including staying physically and mentally active [20]. A way to remain active with low risk of catching different diseases is telehealth. It was tested for patients with ILD during the pandemic and found to be largely well received but did not always meet the needs particularly when they were unwell [21].

Another limitation is that all participants were recruited from the same university hospital. By being able to include patients from smaller hospitals could have broadened the experiences given by the participants during the interviews.

## Conclusions

This is the first study to present results of the experiences of living with idiopathic pulmonary fibrosis (IPF) in relation to physical activity. IPF was experienced by the participants as a disease that constrained physical activity as well as life more generally. Both existential thoughts of how life changed and affected the possibilities of physically activity, as well as coping strategies leading to the management of physical activity were described. It is important that the healthcare system facilitates opportunities for this patient group to maintain an identity of being active persons through the performance of physical activity. Creating coping strategies around physical activity and daily life without assistance can be difficult, and support systems that meet different needs during different phases of the disease should be provided by the healthcare system.

### Electronic supplementary material

Below is the link to the electronic supplementary material.

Supplementary Material 1

## Data Availability

The datasets used and analysed during the current study available from the corresponding author on reasonable request.
