# Supplementary material for: Experiences of living with idiopathic pulmonary fibrosis in relation to physical activity - “How the hills became steeper and steeper”: a qualitative interview study
Source: BMC Pulm Med. 2024 May 23;24:255. doi: 10.1186/s12890-024-03064-z (PMC11118104; doi:10.1186/s12890-024-03064-z)
Supplement: Supplementary file 1 — Supplementary Material 1 [file 12890_2024_3064_MOESM1_ESM.pdf]

Interview guide

Question areas
Physical activity - Activity/Participation - Environmental factors - Quality of life

Debut
• Can you tell us about when you were diagnosed/became ill with pulmonary fibrosis?
• What symptoms did you/are you experiencing?

Activity/participation
• Can you tell us about activities in everyday life or in your daily life that have been affected since you contracted pulmonary fibrosis?

Physical activity
• Can you tell us about your experience of physical activity since you contracted pulmonary fibrosis?
• How has the disease affected your ability for physical activity?
• Have you had to adjust your physical activity since contracting pulmonary fibrosis? If yes, how have you adjusted it?

Environmental factors
• Have the demands from the environment regarding physical function changed since you contracted pulmonary Fibrosis?

Quality of life
• Is there something you avoid or can't do since your illness?

• Has your self-image/quality of life regarding physical function changed after contracting pulmonary fibrosis, and if so, how?

Additional questions used through the interviews:
Is there something you want to tell us more about? / Can you elaborate on this?
